# The proxy dilemma: Informed consent in paediatric clinical research ‐ a case study of Thailand

**DOI:** 10.1111/dewb.12341

**Published:** 2022-01-24

**Authors:** Sheila Varadan, Salin Sirinam, Kriengsak Limkittikul, Phaik Yeong Cheah

**Keywords:** children's rights, informed consent, legally authorised representative, pediatric clinical trials, proxy, research ethics

## Abstract

Informed consent is an essential requirement for the ethical conduct of research. It is also a necessary requirement for the lawful conduct of research. Informed consent provides a legal basis to enrol human subjects in clinical research. In paediatric research, where children do not generally enjoy a presumption of competence, a legal representative must authorise a child's enrolment. Determining who should act on behalf of the child is a matter of law, rather than ethical principle. But, if national laws are lacking or do not reflect socio‐cultural realities, legal uncertainty can arise, which can have implications for children's enrolment in clinical research. Using Thailand as its case study, this paper contemplates how international legal frameworks, such as the UN Convention on the Rights of the Child, could be leveraged to navigate legal uncertainty in the informed consent process, enabling more children to access and participate in paediatric clinical research.

## 1 INTRODUCTION

In 1964, the World Medical Association adopted a set of guidelines for human subject research, in what would become the foundational framework for the ethical oversight of medical research. The Declaration of Helsinki^1^ superseded the Nuremberg Code in scope and content.^2^ It introduced a concept of proxy informed consent,^3^ breaking from the absolute requirement of voluntary and informed consent in human subject research, and paving the way for the ethical conduct of medical research in all categories of persons, including children.

At the crux of informed consent in children is the role held by the proxy–the legally competent adult who holds an ethical duty to safeguard the interests of the child participant, and a legal responsibility to authorise their enrolment in clinical research.

Yet, the role of proxy is not explicated under international and regional ethical guidelines. The Declaration of Helsinki does not elaborate on the proxy decision‐making role nor does it provide a framework to determine who should act as proxy. Over the course of its eight revisions, the Declaration has employed diverse terminology and ascribed different levels of decision‐making authority to the proxy. In earlier versions, it called on a ‘responsible relative'^4^ to give permission replacing ‘that of the [child] subject'^5^, while in later versions it permitted only the ‘legally authorized representative’^6^ to provide informed consent with an understanding that a child's dissent should be respected^7^ and where possible a child's assent should be obtained^8^.

Comparing current international and regional guidelines, there remain notable differences in the terminology and decision‐making authority ascribed to the proxy. The terminology ranges from ‘legally authorized representative’^9^, ‘legally acceptable representative’^10^, ‘legally designated representative’^11^, ‘legal guardian’^12^, ‘parent’^13^ and ‘representative’^14^ with decision‐making authority varying between ‘permission’,^15^ ‘authorisation’^16^ and ‘informed consent’^17^. Determining who should act as proxy is only vaguely discussed, with wide deference given to the ‘applicable laws’ in the jurisdiction of the clinical study.

This legal ambiguity can have practical implications for children's enrolment in paediatric research,^18^ particularly in lower‐ and middle‐income countries^19^ where national laws may be lacking^20^; regulatory oversight remains weak; and socio‐cultural realities of family care do not align with international guidelines.^21^ As Cheah and Parker (2014) explain, ‘[r]egulations for research in children in developing countries are often rigid, confusing or non‐existent.'^22^ Moreover, research ethics committees ‘rarely offer clear guidelines for research in children', and those that ‘adopt international guidelines, with the noble intention of protecting children', often do so with ‘little reflection on their relevance to the local setting, resulting in practical problems for the conduct of the research.’^23^ In these settings, the legal uncertainty, coupled with vague international guidelines become barriers, preventing children from accessing research rather than protecting them through research.^24^ Bwakura‐Dangarembizi et al. (2012) recount their experiences in Zimbabwe, where there are no specific laws on paediatric clinical research: ‘a substantial number of potential research participants were orphans…whose relatives wanted them to be involved in the study but could not because of the requirement…for consent from a parent or legal guardian.’^25^

Focusing on Thailand, where there is currently no law on human subject research and no specific regulations on informed consent in children, this paper examines two areas of legal uncertainty, which commonly arise in the enrolment of children in clinical research: (1) who should act as the ‘legally acceptable representative’ for the child; (2) how informed consent should be obtained in children without a legally recognised representative– children of minor parents^26^, children of parents without legal status, and children living without parental care. It suggests that international legal instruments, such as the UN Convention on the Rights of the Child (CRC), could be leveraged to navigate legal uncertainty in informed consent, providing a framework that not only takes into account children's socio‐cultural environment, but also their right to guidance in the informed consent process in paediatric clinical research.

## 2 BACKGROUND–THE REGULATORY FRAMEWORK FOR INFORMED CONSENT IN PAEDIATRIC CLINICAL RESEARCH IN THAILAND

As in many lower‐ and middle‐income countries^27^, there is no specific law on human subject research, and no regulations directly addressing informed consent in paediatric clinical research in Thailand. Instead, legal codes, statutes and regulatory notifications are pieced together to create an ethico‐legal framework for the informed consent process in paediatric clinical research. However, because it falls on individual ethics committees to lead this process on a case‐by‐case basis, there is a degree of uncertainty and inconsistency in how legal requirements are interpreted and applied across the informed consent process in paediatric clinical research in Thailand.

Advisory guidelines have been developed to support ethics committees in the ethical oversight of clinical research. The Forum for Ethics Review Committees in Thailand (FERCIT)–a coordinating body for ethics committees established in 2000^28^ ‐ developed *Ethical Guidelines for Research* in 2007. The National Research Council of Thailand (NRCT), the main institutional body overseeing all research in Thailand, issued *National Policy and Guidelines for Human Subject Research* in 2015.^29^ While both of these guidelines are useful for the ethical oversight of clinical research generally, neither of them provides guidance on the specific legal requirements for informed consent in paediatric clinical research. In 2012, FERCIT began a process to develop guidelines for paediatric research, issuing the *Ethical Guidance for Research involving Children* in 2015. The 2015 FERCIT guidelines go further in their guidance on informed consent than any previous guidelines.^30^ However, in the absence of a specific law on human subject research, there remain conflicting approaches on how Thai domestic law should be interpreted and applied in the paediatric clinical research setting.

## 3 DISCERNING WHO IS THE LEGAL REPRESENTATIVE‐‘LAR’

Both the *National Health Act B.E.2550 (2007)* and the *Mental Health Act B.E. 2551 (2008)* require written informed consent for medical treatment. The *Mental Health Act B.E. 2551 (2008)* further adds, ‘where the patient is less than eighteen years of age…[a] protector, curator, guardian or a person who takes care of that person, as the case may be, shall give consent…on his behalf.’^31^ The *Mental Health Act B.E. 2551 (2008)*, however, does not provide a legal definition for the persons designated as ‘protector, curator, guardian’ nor does it reference one of the six Thai legal codes as a basis to define the proxy. Apart from the *Mental Health Act B.E. 2551 (2008)*, there is no other legal code or statute that directly addresses the legal requirements of informed consent in clinical research for adults or children in Thailand. The Food and Drug Administration, embedded within the Ministry of Public Health, has regulatory oversight of clinical trials involving the use of drugs in Thailand. The FDA has not issued regulations specific to paediatric clinical research.^32^ However, in its *2013 Clinical Trial Notification*, it specified that all clinical trials must comply with the good clinical practice guidelines issued by the International Conference of Harmonisation (ICH‐GCP).^33^ To this end, the FDA has designated specific ethics review committees to monitor and ensure compliance with the ICH‐GCP guidelines as a condition for regulatory approval for a clinical study.

The ICH‐GCP have issued two sets of good clinical practice guidelines relevant to informed consent in paediatric clinical trials. Under the ICH‐GCP(E6), the general guidelines on good clinical practice in clinical trials, a legally acceptable representative must provide written consent on behalf of a child (legally incompetent research participant) to authorise a child's enrolment in a clinical study. Deference is given to ‘applicable laws’ in the jurisdiction of the clinical trial to define the scope and meaning of ‘LAR'. Under the ICH‐GCP(E11), the guidelines for the use of medicinal products in children, a legal guardian must provide ‘fully informed consent…in accordance with laws or regulations.’^34^ Notwithstanding the discrepancy in terminology, the ICH‐GCP(E6) and ICH‐GCP(E11) impose similar requirements for the role of proxy: (1) the individual must be authorised under the law to act as the legal representative for the child; (2) the individual must be legally competent and capable of providing informed consent on behalf of the child.

Under Thai law, however, there are conflicting legal frameworks to discern who is responsible for the child, and who is legally authorised to represent the child. Using a series of case studies, we consider the practical implications of this legal ambiguity on children's enrolment in paediatric clinical research in Thailand.

### 3.1 Who is a parent for the purposes of informed consent in children

There are conflicting legal definitions for ‘parent’ under Thai law, which can affect a father's ability to act as a legal representative for his child in paediatric clinical research. Under Thai family law, the biological mother is the presumptive legitimate parent of the child.^35^ A father does not enjoy presumptive parental status on the basis of parentage, but rather on the basis of marriage. In other words, if the mother is married to the father at the time of the child's of birth or the child is born within 310 days after the termination of the legal marriage, the father is presumed to be the legitimate parent of the child.^36^ If the father subsequently marries the mother after the child's birth, he becomes the legal parent of the child on the basis of that marriage.^37^ If, however, the mother and father are unmarried at the time of the child's birth and do not subsequently marry, the father must make a formal application to register his status as the legal parent of the child.^38^ Whether the man is the biological father of the child, cared for or lived with the child since birth, will not be determinative of his legal status as a parent under Thai law. Even where a father's biological link to the child is not contested, in the absence of marriage, a father will need to register his legal status as the child's parent. Moreover, if the mother contests the application, a formal hearing will be required to determine whether the father has a biological link or other legal claim to parent the child.

In contrast, Section 4 of the *Child Protection Act B.E. 2546 (2003)* recognises the parental status of both the ‘father and mother of a child, regardless of whether they are married or not.’^39^ The *Child Protection Act B.E. 2546 (2003)* also recognises parental status on the basis of an ongoing caregiving relationship, acknowledging a range of carers (foster parents, step‐parents, guardians) not biologically linked, yet acting as primary carers for the child.

For its part, the *FERCIT Ethical Guidelines on Paediatric Research* suggest a definition for ‘parent’ that aligns with the *Child Protection Act B.E. 2546 (2003)*, recognising both the father and mother as the child's legal representatives, irrespective of marital status or formal registration. In the absence of a specific law on human subject research or regulations on paediatric clinical research, however, there is no legal basis to favour the *Child Protection Act B.E. 2546 (2003)* over the provisions of Thai family law.

Given the uncertainty of a father's legal status as ‘parent', it is not uncommon for research sponsors, particularly foreign commercial sponsors, to require informed consent from a mother (irrespective of a father's eligibility to consent), to ensure the legality of informed consent. This can result in a child's exclusion from research, even if the child's biological father and primary carer is available and capable of providing informed consent. Consider the following scenario.

Scenario #1: The legal status of fathers

The biological mother provides written informed consent (in the presence of the father) for her son to be enrolled in a clinical trial. The boy is enrolled and the study commences. Mid‐way through the trial, changes in the protocol require a re‐consent process for the child. However, only the child's father is available to provide re‐consent. When the trial staff ask for legal documentation, they discover the father is not married to the child's mother. Unsure if the father is the legal parent of the child, the trial staff attempt to contact the mother, but are unable to reach her. The trial staff then tell the father that he cannot consent for the child unless he is legally registered as the child's parent. The trial staff remove the child from the study.

### 3.2 Who is a guardian for the purposes of informed consent in children

There are conflicting legal definitions and frameworks for determining who is a legal guardian for the purposes of informed consent. Under Thai family law, a legally competent adult may be appointed as legal guardian^40^ through the will of the last surviving parent^41^, or by application to the Court from a relative or the Public Prosecutor.^42^ A legal guardian is generally appointed when a child is without parental care either because both parents have died or one or both parents have been deprived of parental rights and responsibilities–partially or fully–under a legal order.^43^ The guardian becomes the legal representative for the child until he or she becomes ‘*sui juris*’^44^ (either by age or legal marriage).^45^ The role of the legal guardian is thus envisaged to replace a parent, extinguishing their rights and responsibilities, including their authority to act as the legal representative for the child.

In contrast, the *Child Protection Act B.E. 2546 (2003)* offers a broad definition for ‘guardian', which includes adoptive parents, step parents, employers, and any other persons providing care or shelter to the child.^46^ The *Child Protection Act B.E. 2546 (2003)* does not enumerate a formal legal process to establish guardianship over a child; and a person acting as ‘guardian’ for a child under the *Child Protect Act B.E. 2546 (2003)* does not appear to extinguish the rights and responsibilities of the child's legitimate parent.

The *FERCIT Ethical Guidelines on Paediatric Research* propose a definition for ‘guardian’ that encompasses both the formally appointed legal guardian under Thai family law, and persons designated as guardians under the *Child Protection Act B.E. 2546 (2003)*. However, in bringing together formally appointed legal guardians, and guardians informally caring for a child, the FERCIT guidelines introduce more confusion rather than clarity over who is authorised to represent the child for the purposes of informed consent.

To confuse matters further, it is not uncommon for children to grow up in intergenerational households with grandparents acting as primary carers *alongside* parents. Consider the following scenario.

Scenario #2: Intergenerational households–grandparents caring for the child

In northeast Thailand, a grandparent brings his sick grandchild to a community health clinic. When the child tests positive for a parasite, clinical trial staff explain to the grandfather that the child is eligible to participate in a clinical trial. The grandfather is keen to enrol his granddaughter. But, when he is asked to produce legal documentation, the trial staff discover he is not formally recognised as the legal guardian for the child. The grandfather explains that he takes care of his grandchild while his son and daughter‐in‐law work to support the family. The researchers tell the grandfather that given the nature of the study (and the risks), written informed consent must be obtained from at least one parent, preferably the mother. The researchers try to contact the child's mother by phone. She provides verbal consent, but is unable to travel to the study site to provide written consent. The child is not enrolled in the study.

In Thailand, as in much of Southeast Asia, it is not uncommon for children to grow up in the care of grandparents.^47^ Childcare and elderly care are intertwined in a broader system of intergenerational reciprocal family care.^48^ Adult children assume social and financial responsibility for their ageing parents^49^, and in exchange grandparents contribute to the care and upbringing of grandchildren.^50^ As part of this arrangement, parents often leave children in the care of grandparents, while pursuing work outside the home for the financial benefit of the entire family.^51^ According to Knodel et al. (2015) majority of elderly Thais receive some material or financial assistance from their adult children, and at least half contribute to some form of childcare.^52^ Because intergenerational family care is widely accepted as a socio‐cultural norm in Thailand, grandparents seldom seek formal recognition of their role as primary caregivers for their grandchildren. Moreover, in the vast majority of cases, parents are still actively involved in the care and upbringing of their children, albeit remotely, whilst working for the benefit of the whole family. If grandparents were to formalize their status as legal guardians within Thai family law, it could potentially extinguish the rights and responsibility of parents, while not capture the intergenerational dimension of the parenting arrangements. Yet, in the absence of a legally recognised caregiving relationship, the status of grandparents remains unclear for the purposes of informed consent in paediatric clinical research. The FERCIT ethical guidelines acknowledge this quandary: ‘in Thailand, it is not common to go to court to seek an order for guardianship, so it is a problem with whom to get consent.’^53^

Kalabuanga et al. (2016) observed a similar quandary in the Democratic Republic of Congo, noting that the requirement for a legally authorised representative ‘fails to take into due account informal social mechanisms’ which often rely on relatives and community to care for a child in *lieu* of biological parents.^54^ So, ‘when a child is brought to a clinic and is eligible for a trial, a question arises whether the caregiver is legally entitled to consent'.^55^ Vischer et al. (2016) in their study on perceptions of the Good Clinical Practice Guidelines (ICH‐GCP) in sub‐Saharan Africa also observed that it was not uncommon for relatives to care for a child in place of biological parents, making it difficult for trial staff to include such children.^56^ Strode et al. (2018) have highlighted an ethico‐legal tension in South Africa, whereby the *National Health Act* (section 71) recognises only parents and legal guardians for the purposes of consent, while national ethical guidelines permit parental substitutes if certain conditions are met.^57^

This gap between formal legal requirements and socio‐culturally realities can lead to an ethically perplexing outcome, whereby a primary carer holds no legal authority in the informed consent process, while the legal representative holds little or no role in the everyday care of the child.

## 4 DISCERNING THE LEGAL REQUIREMENTS OF INFORMED CONSENT IN CHILDREN WITHOUT A LEGAL REPRESENTATIVE

There are certain categories of children whose particular circumstances pose unique legal challenges to the informed consent process. For instance, children of minor parents, children of parents without legal status, and children without parental care do not have legally recognised representatives to act on their behalf. The lack of ethical guidance to navigate the legal requirements of informed consent for these children has tended to result in their presumptive exclusion from clinical research.

### 4.1 Children of minor parents

International and regional ethical guidelines do not address the informed consent process in children of minor parents. Domestic laws also tend to obscure the distinction between who is the legitimate parent for a child and who is the legal representative for the child. This has implications for children of a minor parent, whose legitimate parent may not be recognised as legally competent to act as a legal representative for the purposes of informed consent. In this regard, determining who should provide informed consent for a child of minor parents becomes an ethico‐legal quandary, both in deciding who is best placed to safeguard the interests and welfare of the child participant, and ascertaining who holds legal status to act on behalf of the child for the purposes of informed consent.

Under Thai family law, a child is a ‘minor', and subject to the authority of a legal representative–a legitimate parent or legal guardian–until he or she becomes ‘sui juris’ (legally independent)^58^. A child becomes ‘sui juris’ when he or she turns 20 years of age, or enters into a legal marriage prior to the age of 20 years.^59^ The age of marriage is 17 years (or the completion of the eighteenth year). However, it is legally possible for a child as young as 13 years old to enter into a legal marriage with Court approval.^60^

This raises the question of who is the legal representative for a child of an unmarried minor. According to Thai family law, a child born of a woman who is not married is the legitimate child of that woman.^61^ However, a legitimate parent can only be the legal representative for a child if the parent is also *sui juris*. In other words, an unmarried minor could not be the legal representative of her child, even if she were the legitimate parent of the child. That marital status should be the sole basis to determine the suitability of a minor parent to provide informed consent for a child raises obvious concerns as to the ethical validity of informed consent, but it also raises concerns for the protection and fair treatment of children of minor parents in clinical research. For instance, a child of a married 14 year‐old mother could be enrolled in a clinical trial on the basis that her mother is presumptively competent as a result of her marriage, whereas a child of an unmarried 19‐year old mother would not be eligible to enrol in a trial, even if her mother demonstrated sufficient maturity, understanding and capacity to consent on behalf of her child. Consider the following scenario.

Scenario #3: Children of minor parents

A toddler (3 years old) arrives at a village health clinic with his 17 year‐old unmarried mother and grandmother. The young mother is soothing her son who has a high fever and is crying. The clinic trial staff tell the mother that her son is eligible to participate in a clinical trial on febrile illness, which will help diagnose and treat the cause of his fever. The mother, who is studying to be a nurse, listens intently and is keen to enrol her son in the study. The grandmother, however, is suspicious of the clinical trial staff and does not want her grandson enrolled. The trial staff are unsure whether to accept the consent of the mother who appears to be the primary carer for her son and better informed on her son's care needs, or to respect the refusal of the grandmother, given the mother's young age. In the end, the child is not enrolled in the study.

De Pretto‐Lazarova et al. (2020) conducted a systematic review of informed consent in children of minor parents, citing an apparent lack of an ‘ethically acceptable approach to the IC [informed consent] process’ in paediatric research.^62^ It may be possible to resolve the ethico‐legal gap through a modification of informed consent requirements, particularly where the research envisages a negligible risk to the child. However, in the absence of any ethical or legal guidance on this point, it is likely that children of minor parents will be presumptively excluded from clinical research, not out of an ethical concern but due to the absence of a legal framework that recognises the role of minor parents in the informed consent process.

### 4.2 Children of parents without legal status

There appears to be no ethical guidance on the informed consent process in children of parents without legal status. In some cases, a child may be living with parents who do not have legal status or standing in the jurisdiction of the clinical research study. In other instances, a child may be part of an ethnic minority or religious group that is persecuted, and as such denied legal standing in the jurisdiction of the clinical research study. In both situations, the parent is not legally recognised to act on behalf of the child in the informed consent process. In the absence of specific laws or ethical guidance on this point, there remains a degree of legal precarity as to whether the parent will be allowed to give informed consent, which can affect the fair treatment of children of parents without legal status. Consider the following two scenarios.

Scenario #4 A: Children of refugee parents

A child, born in a Thai refugee camp, is eligible to enrol in a malaria study. The malaria clinical trial is run by a research institution that also provides health care services for undocumented migrants and refugees living in the area. The research clinic has worked with local government authorities to establish an ethically and socio‐culturally appropriate process for recruiting and enrolling children of parents without legal status. The mother is keen to for her son to join the study and the child is enrolled.

Scenario #4B: Children of persecuted or discriminated ethnic minorities

A child, born in an ethnic hilltribe in north eastern Thailand, is eligible to enrol in a vaccine trial. The clinical trial is being conducted by a foreign commercial sponsor. The mother is keen for her son to join the study. However, when the Trial staff ask for legal documentation, they learn that the mother does not have legal status in Thailand. The child is not enrolled in the study.

A child of parents without legal status is likely to be viewed in the same way as a child living in the informal care of grandparents or relatives. It would fall on the ethics committee or institutional review board to determine when and under what conditions the legal requirements of informed consent could be modified to recognise carers not legally authorised to provide informed consent. Such a decision would likely turn on the nature of the research study ‐ the benefit‐risk ratio, the age of the child participants, and the research sponsor's willingness to deviate from international guidelines. It is important to underscore that a child's exclusion would not necessarily be out of ethical concern, but due to a lack of legal guidance on how to navigate the informed consent process in children without legal representatives. Moreover, any modification to the informed consent process, while enabling a child's enrolment, would not address the broader question of whether all children, as a class of persons, are entitled to the guidance and support of a proxy in the informed consent process to enable their participation in clinical research, particularly where the research holds a prospective medical benefit.

### 4.3 Children living without parental care

Beyond the question of who should act as proxy for a child, is the broader question of when a child should be ethically and legally entitled to provide informed consent in clinical research. For the most part, ethical guidelines have deferred to domestic law to determine when and under what conditions a child will be legally permitted to provide informed consent in medical research. However, in the absence of specific laws on human subject research, there may be differing age‐barriers for adulthood, which can introduce confusion around when a child will be deemed legally capable of providing informed consent in research. As Colom and Rohloff observe, ‘regulations vary significantly from country to country regarding when adolescents can provide legal consent’ and ‘even when legal frameworks allow adolescents to seek contraception services without parental permission', they may still require a legal representative to consent on their behalf to medical research.^63^ Consider the following scenario.

Scenario #5: Children living without parental care

*An 18 year‐old boy is living on the streets in Bangkok. An NGO worker notices the boy is unwell and takes him to a public university‐hospital. The boy is diagnosed with cancer, and is placed on a waiting list for treatment. The oncologist tells the boy that he may be eligible for treatment through a clinical drug trial. However, because he is under 20 years of age, written consent is required from both parents or a legal guardian. The boy tells the oncologist that he was kicked out of his home when he was 13 years old and has been living on his own since. After consulting with the ethics committee and the research sponsors, the oncologist regretfully tells the boy he cannot enrol him in the trial*.

Under Thai law, there are conflicting definitions for a child, and differing age barriers for adulthood. Under the *Child Protection Act B.E. 2546 (2003)*, a child is defined as a person under the age of 18 years but does not include persons legally married before the age of 18 years. Under the *Civil and Commercial Code B.E. 2468 (1925)*, a child ceases to be a minor and becomes *sui juris* 64 when they reach the age of 20 years, or become legally married prior to 20 years of age. Under the *Mental Health Act B.E. 2551 (2008)*, a patient who is 18 years or older and legally competent can provide written informed consent to medical treatment. However, children are not recognised as legally competent until they become ‘sui juris', leaving open the question of whether a child who is 18 years old but unmarried will be seen as legally competent for the purposes of informed consent in medical research.

In the absence of specific legislation establishing a minimum age for informed consent or a framework to assess children's capacity to provide informed consent in medical research, there is no clarity as to when and under what conditions a child will be able to provide informed consent in a research study. This uncertainty has direct implications for children living without parental care, who may be presumptively excluded from a study–not out of ethical concern, but due to the absence of a legally authorised representative and/or legal mechanism to assess their capacity to consent. While it may be possible to obtain a waiver in the informed consent process to enable a child's participation in clinical research, particularly where the anticipated risk in a study is negligible, again, this does not resolve the broader question of whether all children–including those living without parental care–have a right to access the informed consent process through a proxy or on their own, to enable their participation in clinical research.

There have been calls for more pragmatic ethical guidelines for paediatric clinical research, which account for the limited regulatory infrastructure and diverse socio‐cultural realities in LMICs.^65^ However, the legal complexities surrounding informed consent in children are not unique to LMICs. As Lepola et al. (2016) reveal in their comparative study of 27 European countries,^66^ there are considerable differences in national legal requirements on informed consent and assent, which can often lead to considerable time and resources being spent on reconciling regulatory differences in multicentre clinical trials.^67^ Lepola et al. (2021) have developed an ‘Informed Consent and Assent Guide’ as a tool to enhance ethical standards of informed consent practice and engender common practices for informed consent in multinational clinical paediatric trials. Whether such a tool could be developed and implemented in LMICs remains questionable. This is in part because the legal uncertainties arising in the informed consent process in LMICs emanates out of an absence of relevant laws, rather than differences between existing laws. In the last section, we contemplate whether international legal frameworks, such as the UN Convention on the Right of the Child, could offer guidance on informed consent in paediatric clinical research, where domestic laws are absent and ethical guidance is lacking.

## 5 NAVIGATING LEGAL UNCERTAINTY IN INFORMED CONSENT IN CHILDREN

The UN Convention on the Rights of the Child (CRC)^68^ is an international human rights treaty, adopted by the United Nations General Assembly in 1989.^69^ It is said to be the most comprehensive^70^ and widely ratified of all human rights conventions, with 196 States parties agreeing to be bound by its legal provisions.^71^

At the crux of the CRC framework is a conception of the child as an independent rights‐holder, whose voice and agency, even if not determinative, must be respected and listened to by adults exercising influence over their everyday life. It reframes the informed consent process from an entitlement held by the proxy over the child to a right vested in the child with an obligation on those adults around the child–the proxy, researchers and ethics review committees–to provide support and guidance, which enables children's participation in the research setting.

### 5.1 Recognising the common responsibilities of both parents in the informed consent process

A unique feature of the CRC is its respect and support for both parents in the care and upbringing of a child.^72^ Indeed, when the CRC was adopted, it offered more support and assistance to parents than any previous instrument under international law.^73^ Article 2(1) requires States to respect and ensure children's rights without any discrimination, which includes preventing discrimination against a child on the basis of his or her parents. Article 18(1) imposes a legal obligation on States to ‘use their best efforts’ to ‘ensure recognition of the principle that both parents have common responsibilities for the upbringing and development of the child.’ Article 5 enshrines a right for all children to receive appropriate guidance and direction from both parents that is consistent with their evolving capacities in the exercise of rights. Taken together, articles 2(1), 18(1), and 5 provide a framework to recognise the legal authority of both parents–fathers and mothers–in the informed consent process.

### 5.2 Recognising informal carers in the informed consent process

The CRC underscores the importance of ‘family’ and ‘family environment’ for children's realization and enjoyment of rights. The preamble of the CRC recognises ‘family’ as the ‘fundamental group of society’^74^ and the ‘natural environment’^75^ for the child's growth and well‐being. Article 5 recognises the importance of not just parents, but wider family and community involved in the everyday parenting of a child.^76^ The explicit reference to ‘extended family and community’ under article 5 reflects an understanding that family structures and parenting arrangements may not always be formalized within the law, and will often be dictated by socio‐cultural norms.^77^ Applied to the paediatric clinical research setting, the CRC may offer a basis to justify a wider reading of ‘legally acceptable representative,’ that takes into account the role of extended family where it is provided for by local custom. In this regard, and in the absence of direct legislation on paediatric clinical research, the CRC framework could offer guidance, acknowledging a child's right to receive support and direction from a wide range of informal carers within the extended family, if such caregiving arrangement are accepted within the community. Such an approach would not only enable practical solutions but also support socio‐culturally appropriate practices in the ethical conduct of research.

### 5.3 Recognising the child's right to guidance and direction in the informed consent process

The CRC recognises that all children, even very young children, are rights‐holder entitled to guidance and direction that supports and respects their developing capacities in the exercise of rights.^78^ The UN Committee on the Rights of the Child has said the concept of ‘evolving capacities’ should act as an enabling principle, requiring adults to provide guidance that not only compensates for a child's lack of knowledge, experience and understanding but also supports the child's capacities to the maximum extent possible.^79^ The CRC framework thus rejects a binary framework for legal competency, recognising that a child's capacities–physical, cognitive, moral, social, emotional and spiritual–will be acquired in a dynamic and fluid process, influenced by genetic, cultural, social and environmental factors.^80^ In the context of paediatric clinical research, this would mean that children without legally authorised representatives would not be presumptively excluded from research, but rather assessed for their actual capacity to give informed consent, and then where necessary, provided with appropriate guidance to enable their participation in the informed consent process. In the case of children of minor parents, it would require a process that respects both the minor parent's ‘capacity rights’ to provide consent for their child, and the child's rights to guidance and protection that enables their participation in the informed consent process in clinical research.

It is important to clarify, that we are not proposing that the CRC be used as a direct substitute for national laws and regulations on paediatric clinical research. As with all international instruments, the CRC will generally not translate into national law, unless a State party takes direct measures to incorporate and implement its legal obligations into domestic law, policy and jurisprudence.^81^ As Kilkelly, Lundy and Byrne (2021) observe, how a State chooses to implement the CRC, and the measures it takes in this regard will have a direct bearing on how it complies with and supports children's rights.^82^ As such, the degree to which the CRC will able to function as a framework to navigate the legal uncertainties of informed consent in paediatric research will depend in some part on what measures the State has taken–legal and non—legal–to incorporate and implement the CRC.^83^ That said, with every country in the world (except the United States of America) having agreed to be legally bound by the provisions of the CRC, it offers the prospect of a common framework to negotiate the legal uncertainties in informed consent in a manner that accords respect and protection to children's rights in the paediatric clinical research setting.

## 6 CONCLUSION

Conducting clinical research in children is ethically and legally complex.^84^ Part of that complexity emanates out of the legal ambiguities surrounding the role of proxy in the informed consent process. This uncertainty is compounded by diverse socio‐cultural realities surrounding parentings and family, which in most parts of the world involves carers within the extended family and wider community. The aim of this paper was to unravel some of that legal complexity by demonstrating the degree of uncertainty that can arise when there are no direct laws or regulations relating to informed consent in paediatric clinical research. It contemplated how international legal frameworks, such as the UN Convention on the Rights of the Child, could be leveraged to negotiate these legal uncertainties in a manner that respects the child's right to access and participate in informed consent in paediatric clinical research. By placing the child at the centre of the decision‐making process, the CRC offers a framework that accommodates a diversity of socio‐cultural environments, while also recognising the child's right to receive guidance and direction in the informed consent process.

However, more research is needed to better understand the implications of legal uncertainty in informed consent and its impact on children's recruitment and enrolment in paediatric clinical research. A more comprehensive comparative legal study is needed on informed consent laws for children in LMICs, which not only identifies the legal gaps but also delves into best practices that have been developed to enable children's access and participation in informed consent. Finally, further research is needed to explore how international legal frameworks, such as the CRC, could be practically applied in the everyday research setting, assessing the challenges and benefits of using human rights frameworks alongside ethical guidelines.

## CONFLICT OF INTEREST

None to declare.

## Footnotes

1 World Medical Association. Declaration of Helsinki: Recommendations guiding doctors in clinical research. Adopted by the 18^th^ World Medical Assembly, Helsinki, Finland, June 1964.

2 Katz, J. (1992). The consent principle of nuremberg: Its significance then and now. In G. Annas, & M. Grodin (Eds.), *The nazi doctors and the nuremberg code: Human rights in human experimentation* (pp. 227–239). Oxford University Press; see also Nuremberg Code, *United States of America v Karl Brandt* et al., 21 November 1946–20 August 1947, judgement reprinted in G. Annas and M. Grodin (Eds), *The nazi doctors and the nuremberg code: Human rights in human experimentation* (pp. 61–144). Oxford University Press.

3 Declaration of Helsinki, 1964, op. cit. note 1, Part II, principle 1 and Part III, principle 3a.

4 World Medical Association. Declaration of Helsinki. (1975). Revised by the 29^th^ World Medical Assembly, Tokyo, Japan, October 1975 (Principle 11); World Medical Association. Declaration of Helsinki. (1983). Revised by the 35^th^ World Medical Assembly, Venice, Italy, October 1983 (Principle 11); World Medical Association. Declaration of Helsinki. (1989). Revised by the 41^st^ World Medical Assembly, Hong Kong, September 1989 (Principle 11); World Medical Association. Declaration of Helsinki. (1996). Revised by the 48^th^ General Assembly, Somerset West, Republic of South Africa, October 1996 (Principle 11).

5 Ibid.

6 World Medical Association. Declaration of Helsinki. (2000). Revised by the 52^nd^ WMA General Assembly, Edinburgh, Scotland, October 2000 (Principle 24); World Medical Association. Declaration of Helsinki. (2004). Revised by the 55^th^ WMA General Assembly, Tokyo, Japan, October 2004 (Principle 15); World Medical Association. Declaration of Helsinki. (2008). Revised by the 59^th^ WMA General Assembly, Seoul, Korea, October 2008 (Principle 27); World Medical Association. Declaration of Helsinki. (2013). Revised by the 64^th^ WMA General Assembly, Fortaleza, Brazil, October 2013 (Principle 28).

7 Declaration of Helsinki, 2008; Declaration of Helsinki, 2013.

8 Declaration of Helsinki, 2000; Declaration of Helsinki, 2004; Declaration of Helsinki, 2008; Declaration of Helsinki, 2013.

9 Declaration of Helsinki, 2013. Principles 28 and 29; Council for International Organizations of Medical Sciences (CIOMS) in collaboration with the World Health Organization (WHO). International Ethical Guidelines for Health‐related Research Involving Humans. Geneva: CIOMS, 2016. Guideline 17: Research involving Children and Adolescents.

10 International Council for Harmonisation of Technical Requirements for Pharmaceuticals for Human Use (ICH): Guidelines for Good Clinical Practice E6(R2). November 9, 2016. Section 1: Glossary, paragraph 1.37.

11 European Parliament. Regulation (EU) No 536/2014 of the European Parliament and of the Council of April 16, 2014 on clinical trials on medicinal products for human use, and repealing directive 2001/20/EC. Article 29; Article 2, para 20.

12 International Council for Harmonisation of Technical Requirements for Pharmaceuticals for Human Use (ICH): Guidelines for Clinical Investigation of Medicinal Products in the Pediatric Population E11(R1). August 1, 2017. Section 2: Guidance, paragraph 2.6.3.

13 Ibid. See also CIOMS 2016, op. cit. note 9, Guideline 17.

14 Council of Europe. The Convention for the Protection of Human Rights and Dignity of the Human Being with regard to the Application of Biology and Medicine: Convention on Human Rights and Biomedicine (ETS No 164). Adopted April 4, 1997. Entered into force December 1, 1999. Article 6 (Oviedo Convention); Council of Europe. Additional Protocol to the Convention on Human Rights and Biomedicine, concerning Biomedical Research (ETS No 195). Adopted January 25, 2005. Entered into force September 1, 2007. Article 15(iv).

15 CIOMS 2016, op. cit. note 9, Guideline 17.

16 Oviedo Convention 1997, op cit. note 14, Article 6; Additional Protocol 2005, op. cit. note 14, Article 15(iv).

17 Declaration of Helsinki 2013, op. cit. note 6; ICH‐GCP (E6) 2016, op. cit. note 10; ICH‐GCP (E11) 2017, op. cit. note 12; EU Regulations 2014, op. cit. note 11.

18 Colom, M., & Rohloff, P. (2018). Cultural considerations for informed consent in paediatric research in low/middle‐incomes countries: A scoping review. *BMJ Paediatrics Open, 2*, 1–14.

19 This paper adopts the definition of ‘lower‐ and middle‐income countries’ used by the World Bank. Retrieved 27 October 27, 2021, from https://datahelpdesk.worldbank.org/knowledgebase/articles/906519

20 Kalabuanga, M., Ravinetto, R., Maketa, V., Mavoko, H.M., Fungula, B., Da Luz, R. I., Van Geertruyden, J. & Lutumba, P. (2016). The challenges of research informed consent in socio‐economically vulnerable populations: A viewpoint from the Democratic Republic of Congo. *Developing World Bioethics, 16*(2), 64–69.

21 Council for International Organizations of Medical Sciences (CIOMS), 2021. Clinical research in resource‐limited settings. A consensus by a CIOMS Working Group. Geneva, Switzerland. Appendix 1. Special Populations, 84–85.

22 Cheah, P.Y., & Parker, M. (2014). Consent and assent in paediatric research in low‐income settings. *BMC Medical Ethics, 15*(22), 1–10, 6.

23 Ibid: 6.

24 Joseph, P.D., Craig, J.C., Tong, A., & Caldwell, P. (2016). Researchers', regulators', and sponsors' views on pediatric clinical trials: A multinational study. *Pediatrics, 138*(4), 1–13: e20161171; Vischer, N., Pfeiffer, C., Joller, A., Klingmann,I., Ka, A., Kpormegbe, S.K., & Burri, C., (2016). The good clinical practice guideline and its interpretation–perceptions of clinical trial teams in sub‐Saharan Africa. *Tropical Medicine and International Health, 21*(8), 1040–1048, 1043. https://doi.org/10.1111/tmi.12734

25 Bwakura‐Dangarembizi, M., Musesengwa, R., Nathoo, K.J., Takaldza, P., Mhute, T., & Vhembo, T. (2012). Ethical and legal constraints to children's participation in research in Zimbabwe: experiences from the multicenter pediatric HIV ARROW trial. *BMC Medical Ethics, 13*(17), 1–5, 3,4. https://doi.org/10.1186/1472-6939-13-17

26 The term ‘minor parents’ is used to refer to parents who are not ‘sui juris’ or ‘legally competent’ for the purposes of providing informed consent under Thai law. See Thailand Civil and Commercial Code B.E. 2468. Book I: General Principles, Title II: Persons, Chapter I: National Persons, Part II: Capacity, sections 19, 20.

27 CIOMS 2021, op. cit. note 21; see also US Department of Health and Human Services, Office of Human Research Protections, ‘International Compilation of Human Research Standards', 2020 Edition. Retrieved October 27, 2021 from https://www.hhs.gov/ohrp/sites/default/files/2020-international-compilation-of-human-research-standards.pdf

28 Forum for Ethical Review Committees in Thailand. Background (unofficial translation). Retrieved October 28, 2021, from http://www.fercit.org/about.php

29 National Research Council of Thailand. (2015). *National policy and guidelines for human research 2015*. National Library of Thailand, 1–112, vii.

30 Forum for Ethics Review Committees in Thailand (2014). Ethical Guidelines for Pediatric Research. Bangkok, Thailand. Retrieved October 28, 2021 from http://www.fercit.org/index.php

31 Mental Health Act B.E. 2551 (2008), sections 20, 21.

32 There are no specific guidelines for reviewing drugs for children and currently no regulations on the oversight of clinical trials in children (unofficial translation). Original version retrieved October 28, 2021, from https://he01.tci-thaijo.org/index.php/TJPP/article/view/169678/122040

33 Thai Ministry of Public Health, FDA regulations for the import of drugs for the purposes of clinical research, current as of January 2021. Original version retrieved October 27, 2021, from https://www.fda.moph.go.th/sites/drug/Shared%20Documents/Law05-Bureau-Drug-announced/A20210205-i.pdf

34 ICH‐GCP (E11), op. cit. note 12. Section 2.6.3., ‘Fully informed consent should be obtained from the legal guardian in accordance with regional laws or regulations.'

35 Thailand Civil and Commercial Code B.E. 2468. Book V: Family, Title II: Parent and Child, Chapter I: Parentage, Section 1546.

36 Thailand Civil and Commercial Code B.E. 2468. Book V: Family, Title II: Parent and Child, Chapter I: Parentage, Section 1536.

37 Thailand Civil and Commercial Code B.E. 2468. Book V: Family, Title II: Parent and Child, Chapter I: Parentage, Section 1547.

38 Thailand Civil and Commercial Code B.E. 2468. Book V: Family, Title II: Parent and Child, Chapter I: Parentage, Section 1548. The mother is entitled to contest the application of the father.

39 Child Protection Act B.E. 2546 (2003). Unofficial translation retrieved October 27, 2021 from http://web.krisdika.go.th/data/outsitedata/outsite21/file/CHILD_PROTECTION_ACT_B.E._2546.pdf

40 Thailand Civil and Commercial Code B.E. 2468. Book V: Family, Title II: Parent and Child, Chapter III: Guardianship, Section 1587.

41 Thailand Civil and Commercial Code B.E. 2468. Book V: Family, Title II: Parent and Child, Chapter III: Guardianship, Section 1586.

42 Thailand Civil and Commercial Code B.E. 2468. Book V: Family, Title II: Parent and Child, Chapter III: Guardianship, Section 1585‐1586.

43 Thailand Civil and Commercial Code B.E. 2468. Book V: Family, Title II: Parent and Child, Chapter III: Guardianship, Section 1582.

44 The term ‘sui juris’ is used to denote ‘legal competence', see Thailand Civil and Commercial Code B.E. 2468. Book I: General Principles, Title II: Persons, Chapter I: Natural Persons, Part II: Capacity, sections 19, 20.

45 Thailand Civil and Commercial Code B.E. 2468. Book V: Family, Title II: Parent and Child, Chapter III: Guardianship, Section 1585.

46 Section 4, Child Protection Act B.E. 2546 (2003). Unofficial translation retrieved October 27, 2021, from http://web.krisdika.go.th/data/outsitedata/outsite21/file/CHILD_PROTECTION_ACT_B.E._2546.pdf

47 Knodel, J., & Nguyen, M.D. (2015).Grandparents and grandchildren: care and support in Myanmar, Thailand and Vietnam. Ageing & Society. 35(1), 1960–1988, 1963; Knodel, J., & Pothisiri, W. (2015). Intergenerational Living Arrangements in Myanmar and Thailand: A Comparative Analysis. J Cross Cult Gerontol. 30(1), 1–20, 17.

48 Ibid: 1963.

49 Knodel & Nguyen, op. cit. note 47, p. 1963.

50 Knodel J., & Chayovan, N. (2009). Intergenerational relationships and family care and support for thai elderly. *Ageing International, 33*(1), 15–27.

51 Knodel, J., Teerawichitchainan, B., Prachuabmoh, V., & Pothisiri, W. (2015). The situation of Thailand's older population: An update based on the 2014 survey of older persons in Thailand. Bangkok: HelpAge International. Retrieved October 27, 2021 from https://www.helpage.org/where-we-work/east-asia/thailand/; Knodel & Pothisiri, op. cit. note 47, p. 17.

52 Ibid; Knodel, J., & Teerawichitchainan, B. (2018). Grandparenting in developing South East Asia: Comparative perspectives from Myanmar, Thailand and Vietnam. In V. Timonen (Ed.), *Grandparenting practices around the world* (pp 65–88). Policy Press Scholarship; see also Ingersoll‐Dayton, B., Punpuing, S., Tangchonlatip, K., & Yakas, L. (2018). Pathways to grandparents' provision of care in skipped‐generation households in Thailand. *Ageing and Society, 33*(1), 1429–1452; Knodel & Pothisiri, op. cit. note 47, p. 10.

53 FERCIT. Ethical Guidelines for Pediatric Research, op. cit. note 30, Section 2.3.

54 Kalabuanga, et al., op. cit. note 20, p. 66.

55 Ibid: 66.

56 Vischer, et al, op. cit. note 24, p. 1043.

57 Strode, A.E., Singh, P.P., Slack, C.M., & Wassenaar, D.R. (2018). Research ethics committees in a tight spot: Approving consent strategies for child research that are *prima facie* illegal but are ethical in terms of national guidelines. *SAMJ, 108*(1), 828–832, p. 829.

58 Thailand Civil and Commercial Code 2468 B.E., Book V: Family, Title II: Parent and Child, Chapter II: Rights and Duties of Parent and Child, Section 1566.

59 Thailand Civil and Commercial Code B.E. 2468. Book I: General Principles, Title II: Persons, Chapter I: Natural Persons, Part II: Capacity, Section 19.

60 Thailand Criminal Code B.E. 2499 (1956), Book II: Specific Offences, Title IX: Offences Relating to Sexuality, Section 277.

61 Thailand Civil and Commercial Code 2468 B.E., Book V: Family, Title II: Parent and Child, Chapter I: Parentage, Sections 1546; Thailand Civil and Commercial Code 2468 B.E., Book V: Family, Title II: Parent and Child, Chapter II: Rights and Duties of Parent and Child, Sections 1564, 1566.

62 De Pretto‐Lazarova, A., Brancati‐Badarau, D.O., & Burri, C. (2020). Informed consent approaches for clinical trial participation of infants with minor parents in sub‐Saharan Africa: A systemic review. *Plos One, 15*(8); e0237088.

63 Colom & Rohloff, op. cit. note 18, p. 12.

64 Thailand Civil and Commercial Code B.E. 2468. Book I: General Principles, Title II: Persons, Chapter I: Natural Persons, Part II: Capacity, Section 19.

65 Joseph, P.D., Caldwell, P.H.Y., Tong, A., Hanson, C.S., & Craig, J.C. (2016). Stakeholders view of clinical trials in low‐ and middle‐income countries: A systemic review. *Pediatrics, 137*(2); e20152800.

66 Lepola, P., Needham, A., Mendum, J., Sallabank, P., Neubauer, D., & de Wildt, S. (2016). Informed consent for paediatric trials in Europe. *Arch Dis Child, 101*, 1017–1025. https://doi.org/10.1136/archdischild-2015-310001

67 Lepola, P., Kindred, M., Gianuzzi, V., Glosli, H., Dehliner‐Kremer, M., Dalrymple, H., Neubauer, D., Boylan, G.B., Conway, J., Dewhurst, J., & Hoffman, D. (2021). Informed consent and assent guide for paediatric clinical trials in Europe. *Arch Dis Child*, 0:1‐9. https://doi.org/10.1136/archdischild-2021-322798

68 United Nations Convention on the Rights of the Child. Adopted November 20, 1989. Entered into force September 2, 1990, 1577 U.N.T.S. 3 (‘CRC').

69 UN General Assembly resolution, ‘Convention on the Rights of the Child,’ UNGA Resolution 44/25, adopted without a vote, 61^st^ plenary meeting, November 20, 1989.

70 Tobin, J. (2019). Introduction: The foundation for children's rights. In J. Tobin & P. Alston (Eds.), *The UN convention on the rights of the child: A commentary* (pp. 1–20). Oxford University Press.

71 As of June 2021, 196 State parties have ratified or acceded to the UN Convention on the Rights of the Child, United Nations Treaty Collection, Status of Treaties. Retrieved October 27, 2021, from https://treaties.un.org/Pages/ViewDetails.aspx?src=TREATY%26mtdsg_no=IV-11%26chapter=4%26clang=_en

72 Doek, J. (2018). The human rights of children: An introduction (pp. 1–15). In U. Kilkelly & T. Liefaard (Eds.), *International human rights of children* (pp. 12–13). Springer Nature; Tobin J., & Varadan, S. (2019). Article 5: The right to parental direction and guidance consistent with a child's evolving capacities (pp. 159–185). In J. Tobin & P. Alston (Eds.), *The UN convention on the rights of the child: A commentary*. Oxford University Press.

73 See Articles 3(2), 5, 9, 10, 16, 18(1), 18(2), 20, 21(a), 22(2), 23(2), 23(3), 24(2), 27(3), 27(4) and 29(1)(c), 37(c), 40(2)(b)(ii), 40(2)(b)(iii), CRC.

74 Preamble para 4, CRC.

75 Preamble para 5, CRC; Lopatka, A. (1996). An Introduction to the United Nations Convention on the Rights of the Child. Transnat'l L. & Contemp. Probs. 6(2), 251–262, pp 251, 255.

76 Article 5, CRC.

77 Tobin & Varadan, op. cit. note 72, pp. 169–170.

78 Varadan, S. (2020). The role of parents in the proxy informed consent process in medical research involving children. *International Journal of Children's Rights, 28*(3), 521–546.

79 Daly, A. (2020). Assessing children's capacity: Reconceptualising our understanding through the UN convention on the rights of the child. *International Journal of Children's Rights, 28*(3), 471–499; Varadan. S. (2019). The principle of evolving capacities under the UN convention on the rights of the child. *International Journal of Children's Rights, 27*(2), 306–338; Lansdown, G. (2005). *The evolving capacities of the child*. UNICEF Innocenti Research Centre.

80 Tobin & Varadan, op. cit. note 72, p. 173.

81 McCall‐Smith, K. (2019). To incorporate the CRC or not–is this really the question? *The International Journal of Human Rights, 23*(3), 425–441. https://doi.org/10.1080/13642987.2018.1558990; Kilkelly, U. (2019). The UN convention on the rights of the child: Incremental and transformative approaches to legal implementation. *The International Journal of Human Rights, 23*(3), 323–337. https://doi.org/10.1080/13642987.2018.1558974

82 Kilkelly, U., Lundy, L., & Byrne, B. (2021). The convention on the rights of the child: A thematic analysis of the incorporation journey (pp. 333–351). In U. Kilkelly, L. Lundy and B. Byrne (Eds.), *Incorporating the UN convention on the rights of the child into national law*. Intersentia, 2021; Kilkelly 2019, op cite note 81, pp. 332–333.

83 McCall‐Smith, op. cit. note 81.

84 CIOMS 2021, op. Cit. note 21, pp. 84–85.

